# The gender and age differences in the passengers’ thermal comfort during cooling and heating conditions in vehicles

**DOI:** 10.1371/journal.pone.0294027

**Published:** 2023-11-10

**Authors:** Jiyoung Kwak, Chungyoon Chun, Jun-Seok Park, Sanghun Kim, Seokwon Seo

**Affiliations:** 1 Department of Interior Architecture and Built Environment, Yonsei University, Yonsei-ro, Seodaemun-gu, Seoul, Republic of Korea; 2 Department of Architectural Engineering, Hanyang University, Wangsimni-ro, Seongdong-gu, Seoul, Republic of Korea; 3 Hyundai Motor Company, Hyundaiyeonguso-ro, Namyang-eup, Hwaseong-si, Gyeonggi-do, Republic of Korea; Southwest Jiaotong University, CHINA

## Abstract

The thermal physiological and psychological responses in vehicles, influenced by gender and age, play a crucial role in ensuring passengers’ comfort. However, these differences have often been overlooked. This study aims to comprehensively examine passengers’ thermal comfort and investigate gender and age disparities based on their physiological and psychological responses. Experiments were conducted inside a vehicle placed in a climate chamber under cooling and heating conditions, with the collected data subjected to statistical analysis. The findings reveal that males had significantly higher mean skin temperatures in cooling conditions and lower skin temperatures in heating conditions than females. However, overall thermal sensation and comfort did not significantly differ between genders. Interestingly, age-related differences were observed to a limited extent in both conditions. This study provides valuable insights into passengers’ thermal responses in vehicles, considering the factors of gender and age, thereby contributing to a comprehensive understanding of thermal comfort in a vehicle environment.

## Introduction

Numerous studies have employed numerical analysis to understand the physical conditions of the vehicle space and develop models for evaluating and predicting thermal comfort. For instance, one study integrated the mean skin temperature and heat balance formula to create a numerical model that captures the interaction between passengers and the vehicle environment [1]. Another research effort focused on a comprehensive analysis of surface heat conduction and its effects to develop a model for transient heat transfer [2]. Moreover, several studies have introduced thermal comfort models that consider various parameters in transient environments or heat exchange models based on heat gain and cooling capacity within the vehicle [3, 4]. Computational fluid dynamics (CFD) models have also played a prominent role in these investigations. By leveraging CFD models, researchers have calculated environmental conditions, such as airflow and temperature distribution, to determine their effects on thermal comfort [5–8].

While these approaches offer advantages in terms of efficiency and reduced reliance on time-consuming experiments, it also carries the risk of not accurately capturing the actual thermal comfort of the passengers. In light of the ASHRAE standard [9], which defines thermal comfort as a condition of mind that expresses satisfaction with the thermal environment, it is more appropriate to assess thermal comfort by collecting responses from subject experiments. In addition, thermoregulation systems and thermal preferences differ among individuals due to factors such as gender, metabolic rates, and clothing resistance, which are not fully reflected in the numerical analysis of environmental parameters [10].

Along with the numerical analysis, Fanger’s Predicted Mean Vote (PMV) model is commonly adopted to assess thermal comfort in various environments. For instance, one study examined the effect of conditioned airflow rate, air-conditioning outlet location, body vent location, and glass properties on thermal sensation using the PMV model [11]. PMV values were also determined using parameters obtained from thermal manikin measurements or CFD models [12, 13]. Moreover, the environmental parameters such as ambient temperature, radiant temperature, direct solar flux, and physiological responses like thermoregulation, heat loss, and heat transfer were calculated and applied to the PMV model to assess thermal sensation [14, 15]. However, the PMV model is not the most suitable method for assessing thermal sensation in the vehicle, as it was originally developed for uniform and steady conditions based on homogeneous assumptions. The transient environment of the vehicle can compromise the PMV model’s accuracy, as demonstrated in other studies [8, 16].

The Equivalent Temperature (Teq) is another commonly utilized index for assessing thermal comfort within a vehicle. Although the names differ, it shares a similar concept with the Equivalent Homogeneous Temperature (EHT). Nilsson introduced thermal comfort zones for various local body parts based on Teq, enabling thermal sensation estimation for both local body parts and overall comfort [17]. Teq does not take into account human subjective aspects like perception and sensation; however, empirical studies have demonstrated a strong correlation between Teq values and thermal perception. Given the asymmetry of the vehicle compartment, the determination and assessment of local Teq values prove valuable [18, 19]. Nevertheless, it is worth noting that some individuals have raised concerns regarding a key limitation associated with localized variations in sensitive heat. This limitation stems from Teq’s reliance on thermal comfort diagrams that do not consider specific clothing conditions when evaluating thermal sensation [20].

In addition to numerical studies, experimentation is another widely used method in thermal comfort research. Specifically, some studies have conducted subject experiments as a complementary approach to validate the models based on numerical analysis. For example, a model for predicting thermal comfort during spot cooling conditions was developed, and its predictions were compared to actual thermal comfort responses obtained from experiments [21]. Similarly, various models were initially developed based on numerical analysis and subsequently validated through experiments [22–25]. In some cases, subject experiments serve as the primary methodology. For instance, researchers conducted experiments with male undergraduates to investigate the effect of heating devices and air intake settings on passengers’ thermal comfort. The thermal sensation and comfort were evaluated by eight or fewer participants in these experiments [26, 27]. Other studies measured the physiological responses such as brain waves, pulse waves, skin temperature, and core temperature alongside thermal comfort assessments [28, 29]. While some studies involved multiple groups of subjects, the number of subjects in each group was small, with fewer than eight people [30, 31].

However, as mentioned above, conducting experiments with a single group or a small number of subjects often overlooks the differences in thermal physiological and psychological responses based on gender and age. Numerous studies have addressed gender and age differences in thermal comfort. Previous research indicates that females are more dissatisfied with a given thermal environment than males, are more sensitive to changes in the optimal thermal environment, and feel less comfortable in cooler conditions [32]. Besides, females showed more significant differences in temperature between the upper and lower bodies than males in cold environments [33]. Physiological and psychological responses also vary with respect to age. With an increase in age, the ability to control body temperature decreases, resulting in older people having a lower heat balance and preferring a warmer environment than younger people [34]. Moreover, compared to young males, middle-aged males had higher skin temperature in winter and lower skin temperature in summer [35]. While numerous studies, including those mentioned earlier, have explored thermal comfort concerning age and gender within controlled environments of climate chambers or real environments such as dwellings, offices, and schools, it is important to note that the environment of a vehicle presents distinct differences. Within a vehicle, local environmental conditions exhibit significant variability due to factors such as exposure to the external environment, which undergoes rapid changes, a large window area, and the proximity of passengers to heating and cooling vents. These unique environmental features exert direct influences on passenger comfort. Heat transfer occurs through multiple mechanisms, including convection, conduction, radiation, and physiological processes such as perspiration and breathing [36]. The intricate interactions of convection, conduction, and radiant heat exchange, instigated by solar radiation, play a substantial role in influencing passenger comfort and the broader vehicle environment. Thus, it is imperative to investigate further the impact of age and gender on thermal comfort in the unique context of vehicle environments.

Therefore, to overcome the limitations of previous studies and gain a comprehensive understanding of the responses of diverse passengers, this study aims to examine the physiological and psychological responses of passengers in vehicles. By examining passengers’ thermal responses based on gender and age, this study is expected to make significant contributions to our understanding of thermal comfort in a vehicle environment.

## Methods

### Experimental conditions

**Fig 1:**
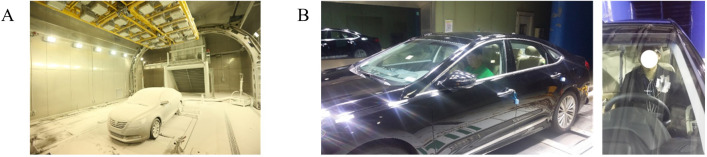
Pictures regarding the experiments. (A) The example picture of the climate chamber. (B) The picture of the subject seated inside the vehicle parked in the chamber.

The experiments were conducted inside a vehicle parked in a climate chamber (Fig 1). Two conditions, cooling and heating, were examined. The chamber and vehicle conditions were strictly controlled to eliminate any environmental differences among the subjects. In the cooling conditions, the climate chamber was adjusted to simulate summer-like conditions, with an ambient temperature of 40°C and solar radiation of 830 W/m^2^, replicating the intense heat experienced during summer. Conversely, for the heating conditions, the chamber was adjusted to winter-like conditions, with a temperature of -20°C and no solar radiation. Fig 1A illustrates the installation of solar radiation panels at the top of the chamber, which provided natural sunlight with global radiation while not directly reaching the subjects’ bodies. The chamber system maintained consistent relative humidity and air speed throughout the experiments, ensuring the desired temperature and solar radiation remained constant.

In the case of the vehicle, the Heating, Ventilation & Air Conditioning (HVAC) system was activated at the start of each experiment. The vehicle was in AUTO mode. In that mode, once the desired ambient temperature was set in the HVAC system, it automatically operated the air conditioner or heater based on the outside temperature. Other environmental parameters, such as relative humidity and air speed, were automatically adjusted to maintain the ambient temperature. This study set a temperature of 23°C for both the cooling and heating conditions. Consequently, the air conditioner was used during the cooling conditions, while the heater was activated for the heating conditions. Unlike the chamber, the vehicle’s indoor environment changed throughout the experiments due to the continuous operation of the HVAC system. So, before each subsequent experiment, the vehicle was fully soaked for over 4 hours to ensure consistent initial conditions. Moreover, each experiment’s environment was meticulously controlled to ensure identical conditions. It was achieved by keeping the chamber environments, which directly influenced the operation of the vehicle’s HVAC system, the same across all experiments.

These experimental conditions, including the environmental settings of the chamber and the vehicle, represent a general evaluation environment for assessing the vehicle’s performance in terms of heating and cooling capabilities across different regions and climates, with a focus on performance under extreme conditions.

Prior to the experiment, subjects spent 20 min in a pre-conditioned room to stabilize their thermal state and metabolism. Although the room environment was not precisely controlled like a climate chamber, efforts were made to provide a similar condition for all subjects. The average temperature and relative humidity of the pre-conditioned room in the cooling conditions were 26.91°C and 20.70%, respectively, while in the heating conditions, they were 22.38°C and 15.64%.

### Subjects

The experiments involved Korean males aged 20 to 39 (2030M), males aged 40 to 59 (4050M), and females aged 20 to 39 (2030F). It is known that the thermal comfort of the subjects may vary depending on their body fat percentage [37, 38]. Therefore, subjects within the normal range of body fats were selected for the study (2030M: 19.8% ±10, 2030F: 29.3% ±10, 4050M: 21.4% ±10, 7th Human Dimension Survey, SizeKorea, 2015).

It is worth noting that the physiological responses in females undergo significant changes before and after menopause. Post-menopausal females tend to have a wider allowable temperature range and often experience sensations of heat, resulting in an average rise of 1°C in skin temperature when feeling hot [39, 40]. For these reasons, females in their forties and fifties were excluded from this study.

According to ISO 14505–3 [41], a minimum of eight subjects shall be selected to assess vehicle thermal comfort. Therefore, more than eight subjects per session were recruited for the study. The number of subjects and their characteristics are provided in Table 1. Different subjects participated in each session in the case of 2030M and 4050M. However, for 2030F, the same subjects were involved in the cooling and heating conditions, resulting in consistent characteristics across the sessions.

**Table 1:** Characteristics of the subjects.

Sessions	Number of subjects	Age	Height (cm)	Weight (kg)	Body Fat (%)
2030M_Cooling	14	29.3 ±4.3	175.5 ±4.6	72.5 ±5.4	18.1 ±3.2
2030M_Heating	19	30.1 ±3.0	176.8 ±3.1	73.0 ±5.0	17.4 ±3.2
2030F_Cooling	20	25.1 ±2.2	163.9 ±4.4	58.0 ±9.1	29.8 ±6.0
2030F_Heating	20	25.1 ±2.2	163.9 ±4.4	58.0 ±9.1	29.8 ±6.0
4050M_Cooling	19	47.4 ±5.4	172.2 ±6.4	77.1 ±9.0	25.7 ±5.0
4050M_Heating	18	47.4 ±5.6	173.1 ±7.0	80.3 ±9.2	25.9 ±4.9

Values represent “mean ± standard deviation”.

The experiments were conducted in 2016, 2018, and 2019. Specifically, the experiments for 2030M took place from August to December 2016, those for 2030F occurred in October 2018, and the experiments for 4050M were carried out from January to May 2019. All experiments were conducted after obtaining the necessary ethical approval from the Institutional Review Board (IRB) of Yonsei University (IRB no. 7001988-201805-HRBR-187-05 for the 2030M experiments, and IRB no. 7001988-202004-HR-407-04 for the 2030F and 4050M experiments). Also, a consent form was obtained from all subjects before starting the experiment.

Despite being conducted in different years, there were no differences in the environmental conditions among the groups, as all experiments took place in the same environment using the same chamber and vehicle. Additionally, before starting the experiments, the subjects’ thermal states were stabilized in the pre-conditioned room during the same time. This approach effectively eliminated any potential effects of varying outdoor conditions, which may exist due to different years among the groups.

All subjects wore the same clothes for the experiments, which were chosen to represent the typical indoor clothes in summer and winter. In the heating conditions, subjects wore long-sleeved T-shirts, jeans, socks, and jackets, as the passengers usually take off their coats and wear lighter clothes after boarding in winter. The clothing value, which indicates the clothing insulation, was 0.85. For the cooling conditions, subjects wore short-sleeved T-shirts, long cotton trousers, socks, and sneakers, resulting in a total of 0.51 clo. These clothing values were obtained through a thermal manikin test at a nationally accredited testing laboratory.

### Measurement

**Fig 2:**
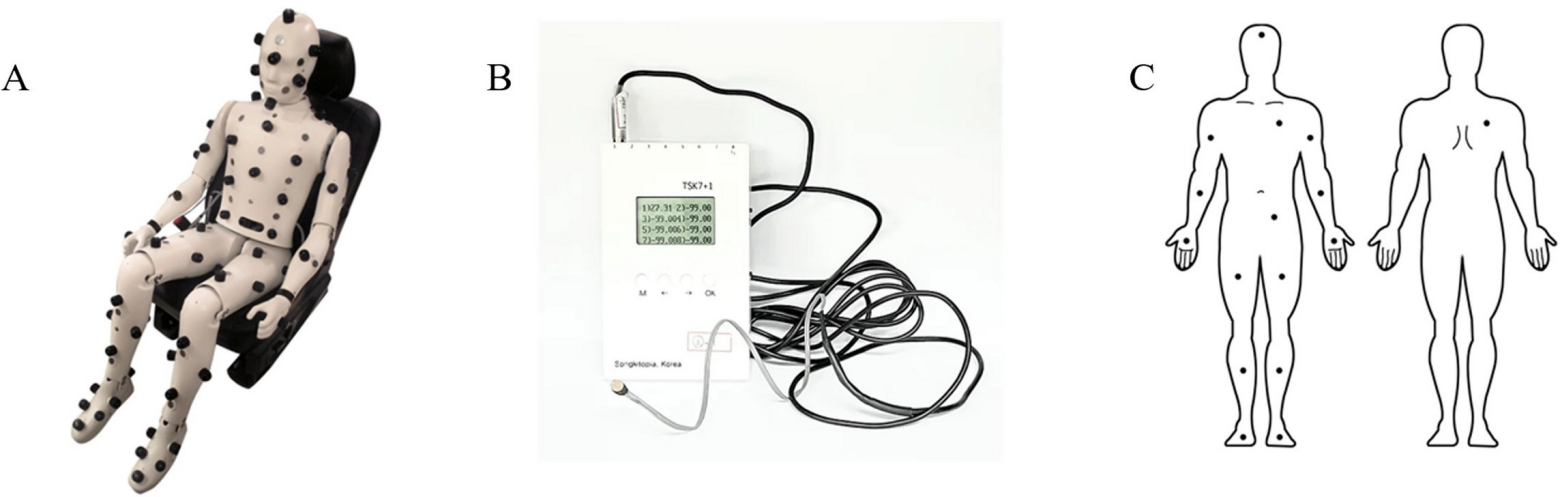
The measuring equipment. (A) The thermal manikin for the environment measurement. (B) The sensor and data logger for the skin temperature measurement. (C) The 16 body parts where the skin temperature sensors were attached.

The environmental parameters were measured separately to avoid interrupting the subjects and the experiments. After completing the entire experiment, measurements were conducted with an HVAC manikin under the same conditions and duration as the experiments. The experiments were carried out under highly controlled conditions, allowing for replicating the same conditions during the measurement phase. An Automotive HVAC manikin (Thermetrics, Seattle, USA) with an accuracy of ±1.0°C and equipped with 60 measuring points throughout the body was used (Fig 2A). The manikin was seated in the driver’s seat, mirroring the positioning of the subjects, and the measurements of ambient temperature, air speed, radiant heat flux, radiant temperature, and relative humidity were taken at each point, enabling monitoring of the environmental conditions by body part.

Regarding the subjects’ thermal physiological responses, skin temperatures were measured using TSK 7+1 sensors (accuracy ±0.1°C, eight-channel, Songkitopia, Korea, Fig 2B). The sensors were attached to 16 parts of the subject’s body, and they continuously measured changes in skin temperatures at 10-second intervals throughout the experiments. The 16 parts included the upper arms, forearms, hands, anterior thighs, shins, feet, chest, abdomen, back, and forehead, as illustrated in Fig 2C. After measuring the skin temperature in these 16 body parts, the mean skin temperature (MST) was calculated using the Hardy & Dubois formula (1) [42] to examine the overall change in the subjects’ skin temperature.


$$0.07\;\times \;\left(T_{Forehead\;}\right)\;+\;0.35\;\times \;\left(T_{Chest\;}\right)\;+\;0.14\;\times \;\left(T_{forearms\;}\right)\;+\;0.07\;\times \;\left(T_{Feet\;}\right)\;+\;0.13\;\times \;\left(T_{shins\;}\right)\;+0.19\;\times \;\left(T_{Thighs\;}\right)\;+\;0.05\;\times \;\left(T_{Hands}\right)$$ (1)

**Fig 3:**
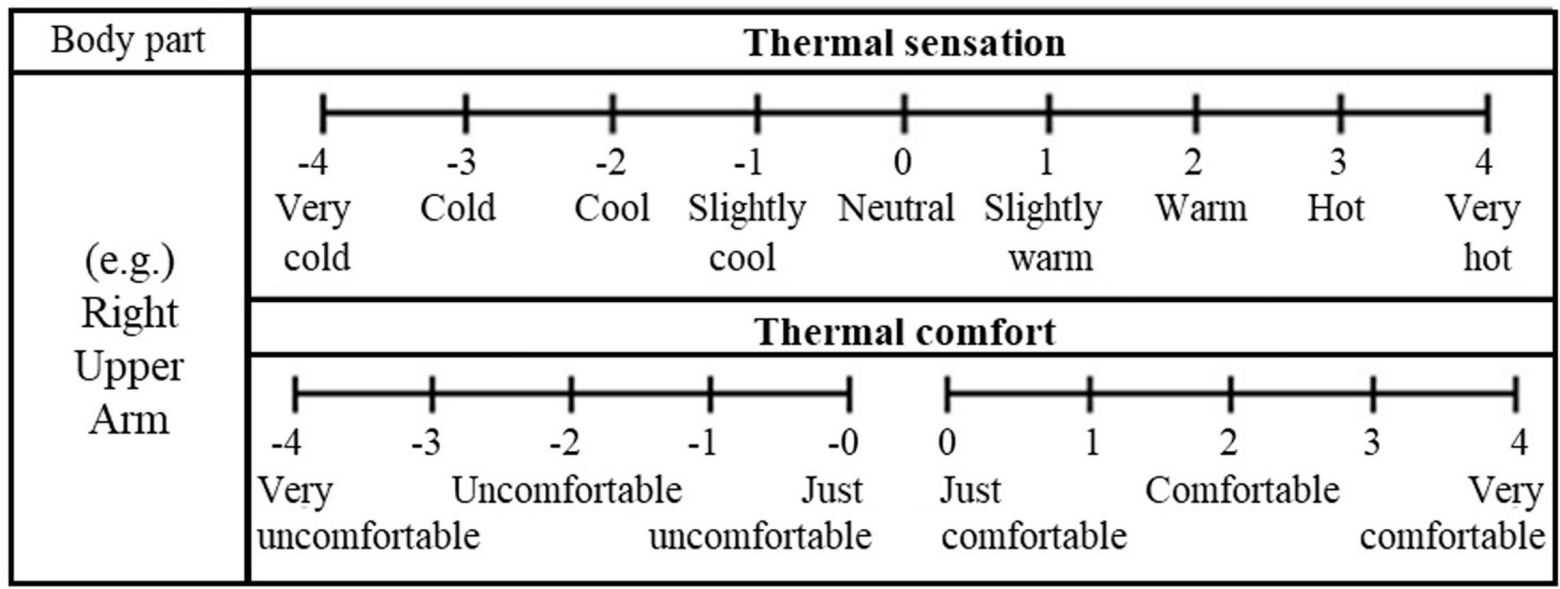
Scales for thermal comfort survey.

To analyze the thermal psychological responses of the subjects, thermal sensation (TS) and thermal comfort (TC) votes were obtained from the subjects. The subjects evaluated their local and overall TS and TC at 5-minute intervals throughout the 60-min experiments, amounting to a total of 13 evaluations. TS was rated on a 9-point scale, while TC was assessed using a centrally separated 10-point scale (Fig 3), following the methodology outlined in a previous study [43]. In the TC scale, scale 0 is further divided into +0 and -0 to facilitate the subjects’ identification of their comfort level. However, for the convenience of analysis, +0 and -0 were combined and treated as just 0.

### Experimental procedure

**Fig 4:**
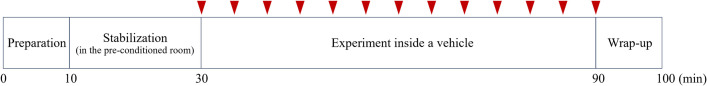
Experimental procedure. (Red triangles indicate the moments when the thermal comfort survey was conducted.).

The experimental procedure is described in Fig 4. This procedure was consistent across all experiments in both conditions. The procedure can be outlined as follows:

1. Preparation: Upon the subjects’ arrival, they signed the consent form. Then, their weight, height, and body fat were measured. After that, they changed into the designated experimental clothes. Additionally, the researcher attached skin temperature sensors to 16 parts of their bodies.
2. Stabilization: The subjects stayed in the pre-conditioned room for 20 min to stabilize their body’s thermal state and metabolism.
3. Experiment: Following the stabilization period, the subjects entered the vehicle parked inside the climate chamber. The measurement of skin temperature commenced, and the experiment officially began. Each experiment had a duration of 60 min, during which the subjects evaluated their local and overall TS and TC at 5-minute intervals. Only one subject was seated in the driver’s seat during each experiment to prevent potential psychological interference due to the presence of other subjects.

### Data treatment and statistical method

The environmental parameters and skin temperatures were recorded in each data logger, and the data was saved in a CSV file after the experiments. Tablet PCs or printed sheets were used for the thermal comfort survey, and the subjects directly marked their votes on the scales. Therefore, coding was necessary to convert the data into numerical values and save it as a CSV file. The environment, skin temperature, and thermal comfort data were then organized by matching the corresponding timestamps. Invalid data resulting from errors during the experiments or treatment were excluded. The total number of valid data used for the analysis is shown in Table 2. Subsequently, an independent samples t-test was conducted using IBM SPSS Statistics Ver 26 to examine gender and age differences. Subsequently, an independent samples t-test was conducted using IBM SPSS Statistics Ver 26 to examine gender and age differences across all experimental conditions. In the manuscript, we have chosen to highlight and present the results that revealed statistically significant differences. For those findings that did not yield any statistically significant differences, we briefly mention them within the relevant context without including detailed statistical tables. Throughout the entire process of data treatment and analysis, the data was used anonymously.

**Table 2:** The number of valid data per session.

Conditions	2030M	2030F	4050M
Cooling	13	18	19
Heating	15	16	16
Total	28	34	35

## Results

### Indoor environment of the vehicle in cooling and heating conditions

**Fig 5:**
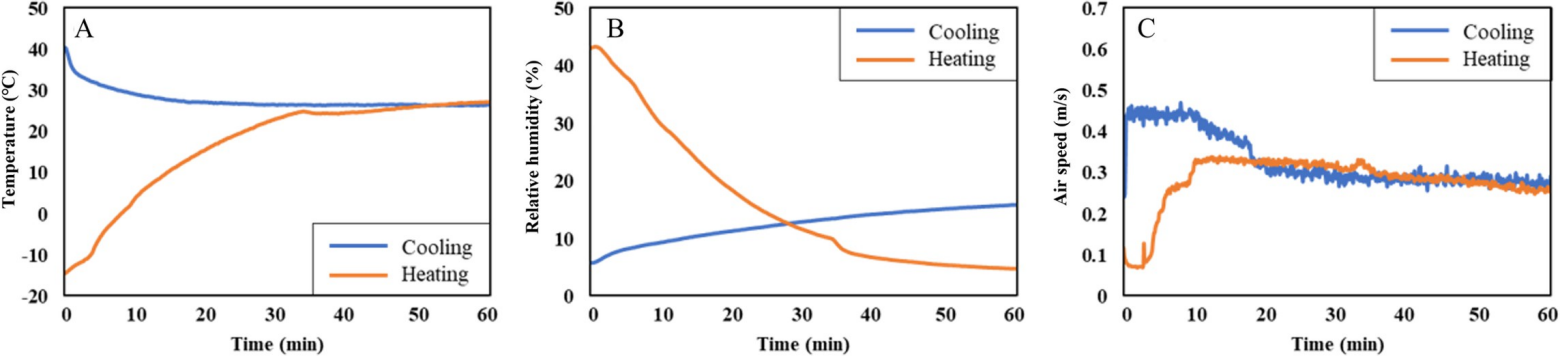
The averaged values of environmental parameters measured from the manikin’s 16 points. (A) The ambient temperature. (B) The relative humidity. (C) The air speed.

The indoor environmental parameters were measured using the HVAC manikin. Out of its 60 measuring points, 16 points were selected and averaged for analysis. These 16 points coincided with the locations where the skin temperature sensors were attached to the subjects’ bodies. The results presented in Fig 5 represent the averaged values of these 16 points per parameter.

Overall, the environmental parameters exhibited significant changes initially and then stabilized over time. Under cooling conditions, the ambient temperature decreased from approximately 40°C to 27°C within 25 min, then remained at around 26.5°C. Conversely, in the heating conditions, the ambient temperature increased from -14°C to 25°C in 33 min and was maintained at 26.5°C after that (Fig 5A). Toward the end of the experiments, the temperature in both conditions converged because the set temperatures of the HVAC system were the same. Although the set temperatures were 23°C, the actual temperature near the subjects was approximately 3°C higher.

Regarding relative humidity (Fig 5B), it continuously increased from 6% to 16% throughout the 60-min duration of the cooling conditions. In contrast, the humidity gradually decreased from 43% to 5% in the heating conditions. Other than ambient temperature, the parameters were automatically adjusted to maintain the ambient temperature at 23°C, leading to substantial changes in the humidity, particularly during the heating conditions. Nevertheless, the subjects did not report any discomfort associated with the humidity, as it is normal to be relatively low inside the vehicle where the humidity controllers do not exist.

Fig 5C illustrates air speed in both conditions. Initially, it was 0.45 m/s, which decreased to 0.3 m/s after 15 min, stabilizing around this value during the cooling conditions. In the heating conditions, the air speed exhibited an initial increase from 0.12 m/s to 0.33 m/s within the first 10 min, followed by a gradual decrease to 0.26 m/s by the end of the experiments.

The solar radiation panel installed on the chamber ceiling had a direct impact on the vehicle. Given the confined space within the vehicle and the proximity of the driver to the vehicle door and vent, it was evident that radiant temperature could deviate from the ambient temperature and vary across local areas. Therefore, radiant temperatures were also observed. Radiant temperatures were measured using an HVAC manikin, and the average values were calculated for specific body parts: "Torso," comprising the chest, abdomen, and back; "Upper body," which encompassed the upper arms, forearms, and hands; and "Lower body," representing the thighs and shins. Radiant temperatures of the feet were not measured.

**Fig 6:**
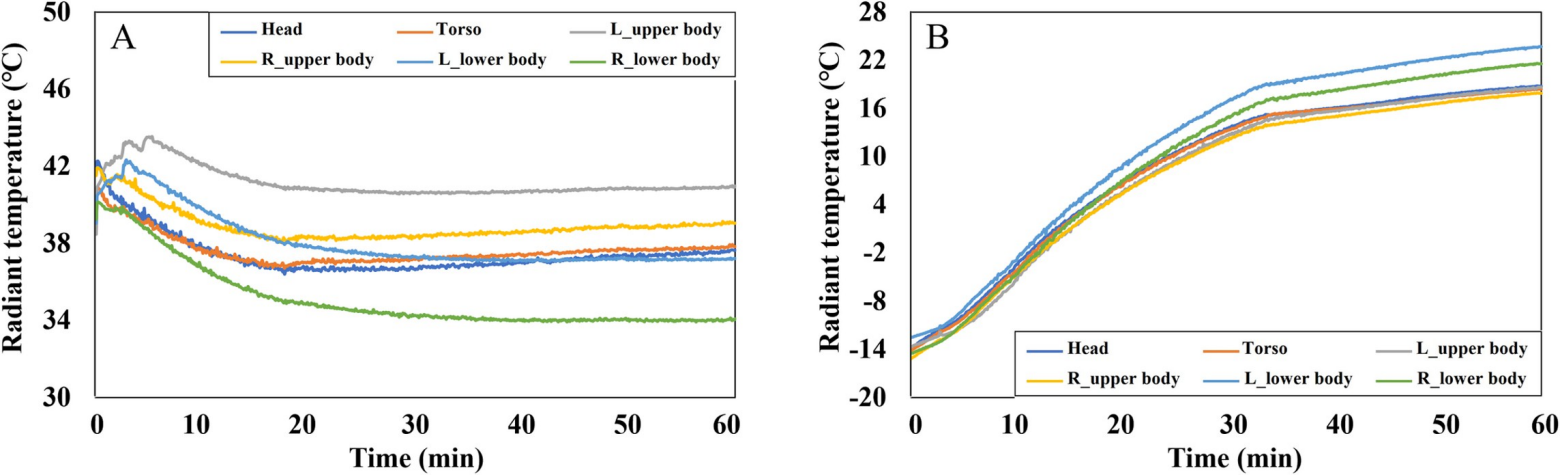
The averaged values of radiant temperatures measured from the manikin’s 16 points. (A) Cooling conditions. (B) Heating conditions.

In the cooling conditions, the ambient temperature decreased from 40 degrees to 27 degrees (Fig 5A). However, when considering radiant temperature (Fig 6A), the overall rate of decrease was smaller than that of the ambient temperature. Consequently, radiant temperatures remained higher than the ambient temperature even after the environmental conditions stabilized. The radiant temperature near the right lower body, furthest from the vehicle door, exhibited the most significant decrease and eventually stabilized at around 34 degrees, approximately 7 degrees higher than the ambient temperature. Conversely, the radiant temperature of the left upper body, situated closest to the window, was the highest. The radiant temperature of that part stabilized at 40.9 degrees, about 14 degrees higher than the ambient temperature.

In contrast, the differences in radiant temperature across local body parts were relatively small in the heating conditions (Fig 6B). This can be attributed to the absence of solar radiation in the heating conditions. Radiant temperatures for all body parts initially started below zero, mirroring the ambient temperature, and steadily increased until 30 min, after which the rate of increase decelerated. In the stabilization phase (after 30 min), the radiant temperature of both lower bodies was the highest, reaching 21.5 degrees on the right and 23.7 degrees on the left at the end. This observation is likely due to the warm airflow directed toward the feet when the heating was activated.

### Results in cooling conditions

#### Overall responses

**Fig 7:**
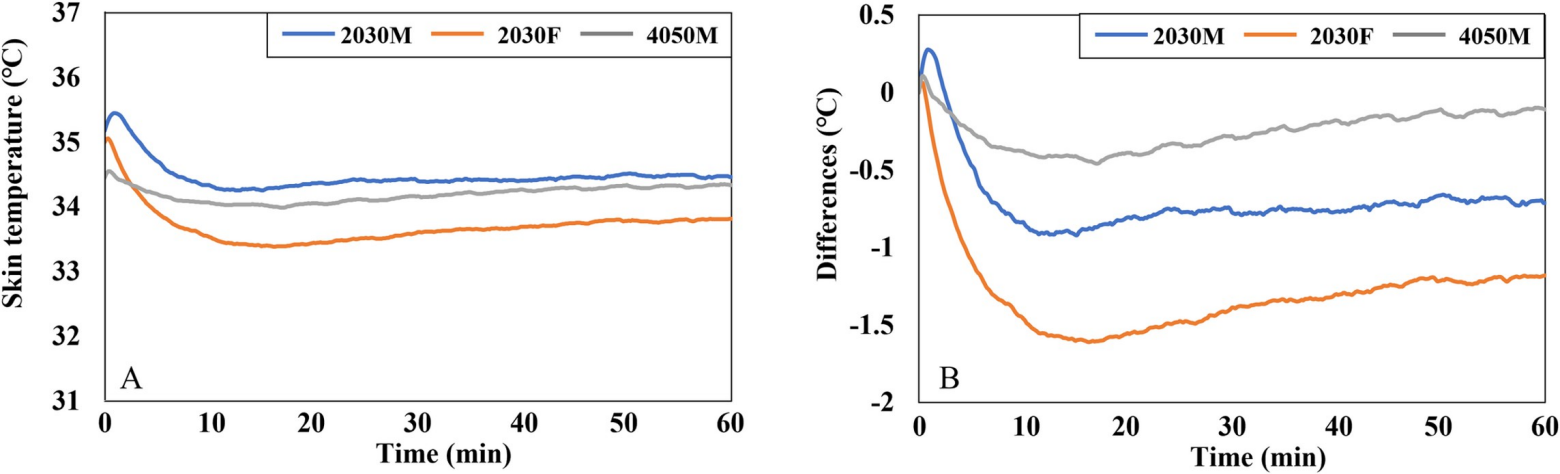
Graphs of MST in the cooling conditions. (A) The MST of three groups. (B) The differences in the MST of each group when compared to the MST at the beginning of the experiment (0 min).

Fig 7A illustrates the MST of three groups. After an initial drop, the MST of all three groups stabilized for the remaining duration. The MST of 2030M started at 35.2°C and stabilized at around 34.4°C. The MST of 2030F decreased from 35°C to 33.4°C within the first 15 min and then stabilized between 33.4–33.8°C. The MST of 4050M remained stable within the 34–34.3°C range.

Fig 7B presents the change in MST over time compared to the beginning of the experiment (0 min). According to the graph, the MST of 2030F exhibited the largest decrease (-1.5°C) compared to the beginning of the experiment (0 min). In contrast, the MST of 4050M showed the smallest decrease (less than -0.5°C).

An independent t-test was conducted using MST samples extracted every 5 min corresponding to the thermal comfort response intervals to examine the differences based on gender and age (Table 3). Throughout the experiment (5–60 min), there were statistically significant gender differences between 2030M and 2030F at a significance level of 0.05. The MST of 2030F was consistently 0.65–0.92°C lower than that of 2030M. However, significant age differences were observed only during the first 5 min, with the MST of 4050M being 0.73°C and 0.51°C lower than that of 2030M at 0 and 5 min, respectively.

**Table 3:** The results of the t-test for gender and age differences in MST in the cooling condition.

Time (min)	Gender difference (2030M-2030F)		Age difference (2030M-4050M)	
	t-value	p-value	t-value	p-value
0	0.96	.344	3.79	.001**
5	4.47	.000**	2.82	.008**
10	4.45	.000**	1.53	.136
15	4.81	.000**	1.33	.194
20	4.88	.000**	176	.089
25	4.62	.000**	1.53	.137
30	4.21	.000**	1.16	.256
35	3.85	.001**	0.89	.379
40	3.71	.001**	0.65	.522
45	3.83	.001**	0.74	.465
50	3.65	.001**	0.77	.447
55	3.54	.001**	0.70	.487
60	3.15	.004**	0.52	.603

**p<0.05 (= The differences are statistically significant.)

**Fig 8:**
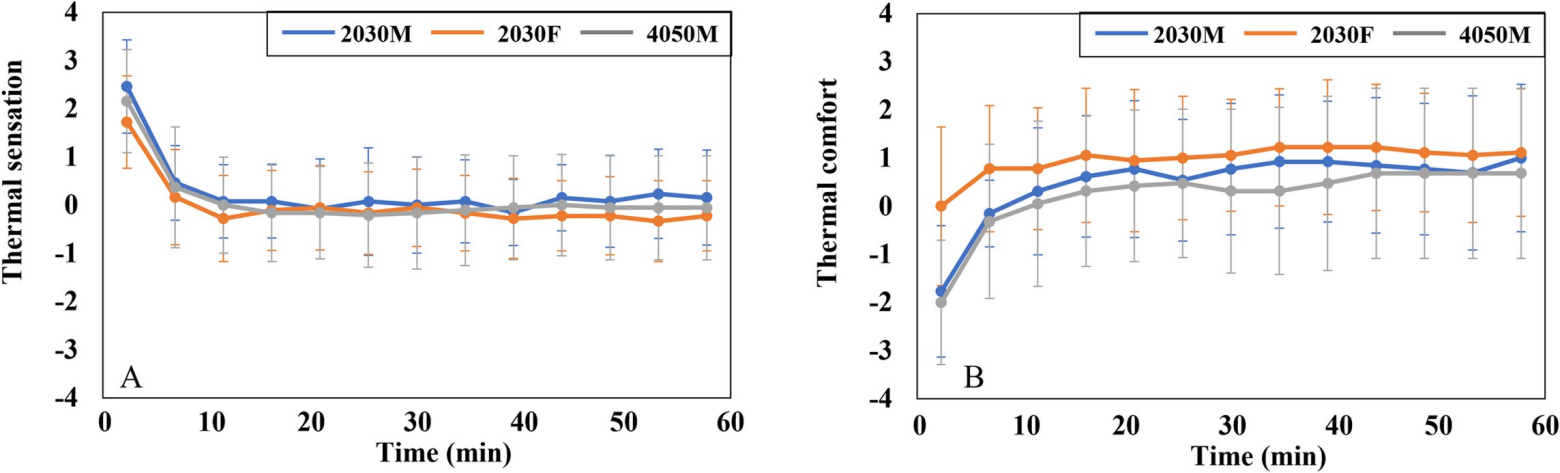
Graphs of psychological responses of three groups in the cooling conditions. (A) The mean overall TS. (B) The mean overall TC. The graphs display the mean values and standard deviations for each variable.

In addition to MST, the thermal psychological responses were analyzed. The overall TS is described in Fig 8A. Initially, all subjects reported feeling hot (scale +2), but their overall TS stabilized at neutral (scale 0) after 10 min. Fig 8B displays the graph of the overall TC. The TC level of 2030F started at scale 0 and then increased to scale +1, maintaining this level throughout the experiment. Both male groups felt uncomfortable (scale -2) at 0 min, but as the overall TS decreased, their TC level rose to scale 0 and remained between scale 0 and +1.

The t-test results for gender differences in overall TS and TC can be found in Table 4. It was observed that there were statistically significant variations in overall TS only at 0 min and in overall TC during 0–5 min. However, as the experiments progressed, significant differences between genders disappeared. Additionally, there were no significant variations based on age, as the overall TS and TC of the two male groups were similar.

**Table 4:** The results of the t-test for gender differences in overall TS and TC under cooling conditions.

Time (min)	Overall TS		Overall TC	
	t-value	p-value	t-value	p-value
0	2.11	.043**	-3.17	.004**
5	0.90	.378	-2.57	.016**
10	1.16	.256	-1.01	.323
15	0.64	.525	-0.90	.374
20	-0.06	.951	-0.33	.743
25	0.69	.497	-0.99	.329
30	0.17	.865	-0.63	.534
35	0.82	.420	-0.64	.528
40	0.44	.663	-0.61	.544
45	1.45	.159	-0.77	.450
50	0.94	.354	-0.73	.472
55	1.77	.088	-0.67	.506
60	1.22	.232	-0.22	.830

**p<0.05 (= The differences are statistically significant.)

#### Local responses

**Fig 9:**
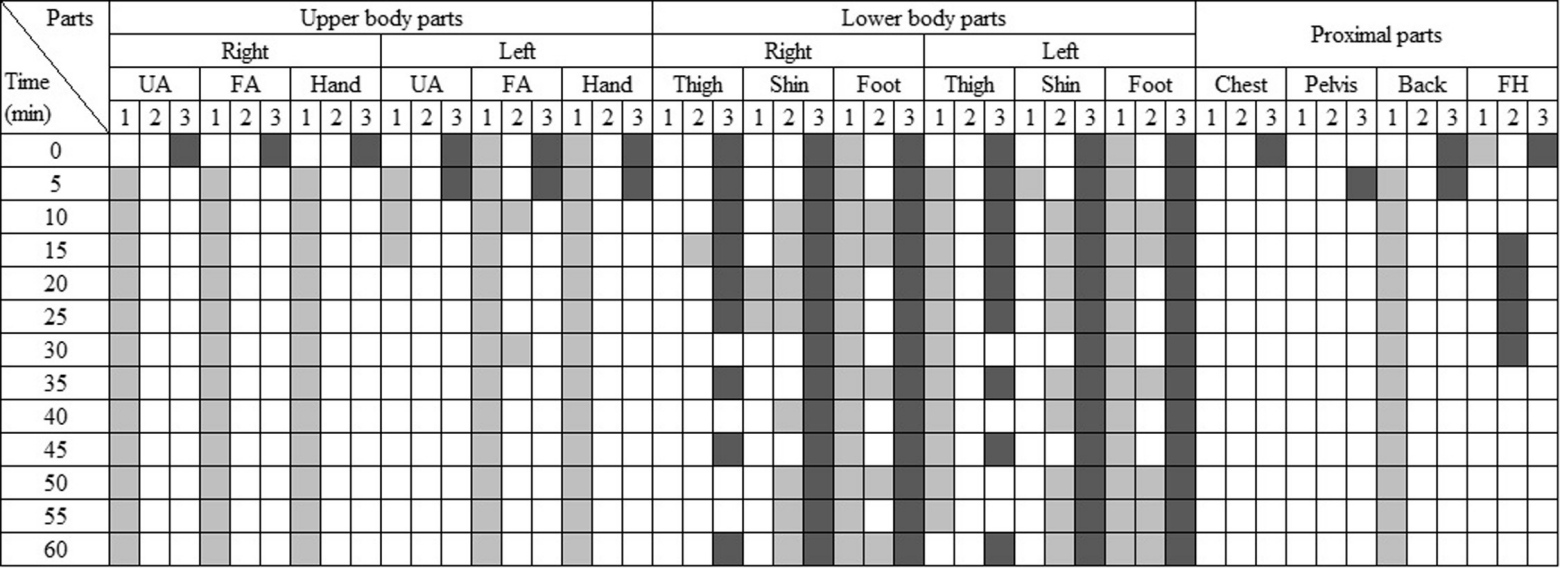
The matrix showing body parts with statistically significant gender differences in the cooling conditions. (p < 0.05. UA: upper arm, FA: forearm, FH: forehead. 1: skin temperature, 2: TS, 3:TC. Light color: 2030M had higher skin temperature, TS, TC than 2030F. Dark color: vice versa).

Alongside the overall responses, the local responses were also examined. Fig 9 illustrates the body parts where gender differences in local skin temperature, TS, and TC were significant at each time interval (p<0.05). Blank cells indicate no significant differences between genders. Light colors indicate that 2030M had higher temperatures, TS, or TC, whereas dark colors indicate that 2030F had higher skin temperatures, TS, or TC.

Generally, differences were observed in skin temperatures at the upper body parts and in TC at the lower body parts. Among the upper body parts, except for the left upper arm, 2030M had significantly higher skin temperatures than 2030F. However, no significant differences were observed in TS and TC, indicating that females experienced similar TC despite having lower skin temperatures. While the skin temperature of 2030M was higher only on the left thigh, 2030F had higher TC than males on both thighs. Additionally, females reported higher comfort levels than males at the shins and feet. It suggests that males felt less comfortable due to higher TS at the shins and higher skin temperatures on the feet. Females experienced elevated TS only in the forehead for 15–20 min, but no differences were observed in skin temperatures or TC.

**Fig 10:**
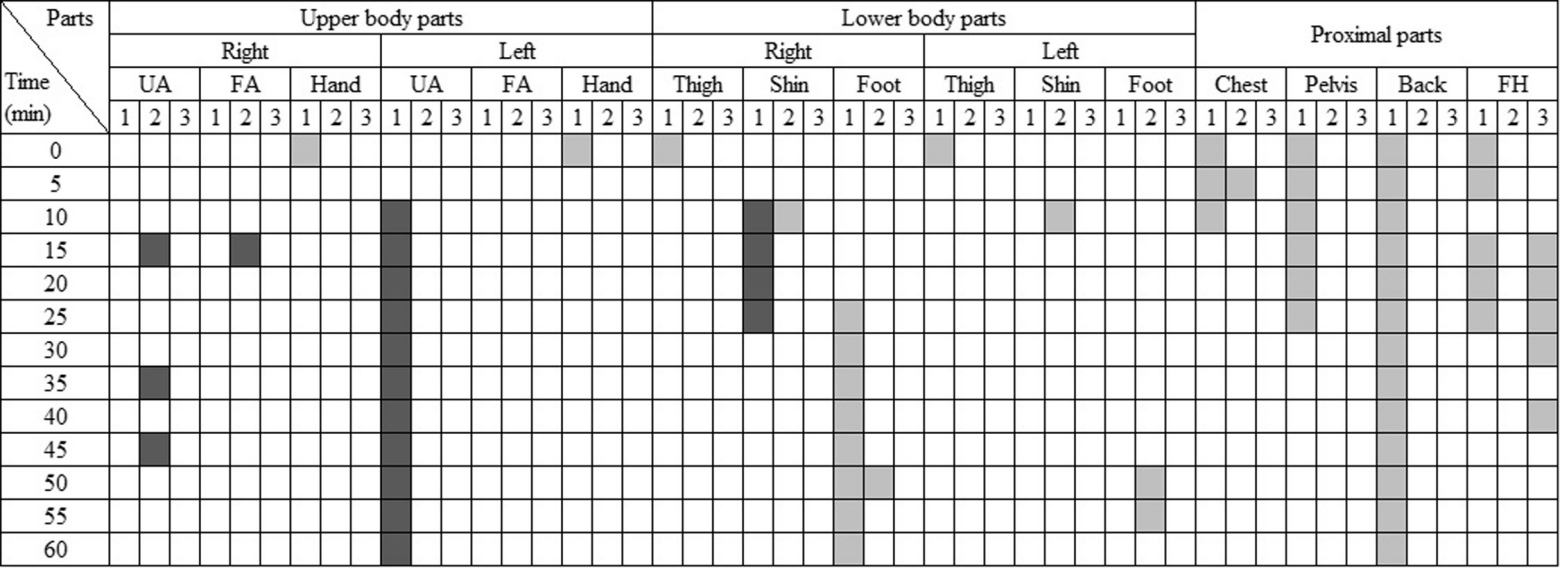
The matrix showing body parts with statistically significant age differences in the cooling conditions. (p < 0.05. UA: upper arm, FA: forearm, FH: forehead. 1: skin temperature, 2: TS, 3:TC. Light color: 2030M had higher skin temperature, TS, TC than 4050M. Dark color: vice versa).

In contrast to the gender differences, no significant variations in local responses were observed based on age (Fig 10). Although 4050M had significantly higher skin temperatures on the left upper arm and right shin, these differences did not result in variations in TS or TC. The higher temperature observed on the left upper arm is likely a result of radiant heat coming through the window or door on the left side. Moreover, while 2030M had higher skin temperatures at the right foot and proximal parts compared to 4050M, no differences in TS and TC were observed in those parts. Interestingly, 2030M reported a higher comfort level at the forehead for 15–25 min despite the high skin temperature.

### Results in heating conditions

#### Overall responses

**Fig 11:**
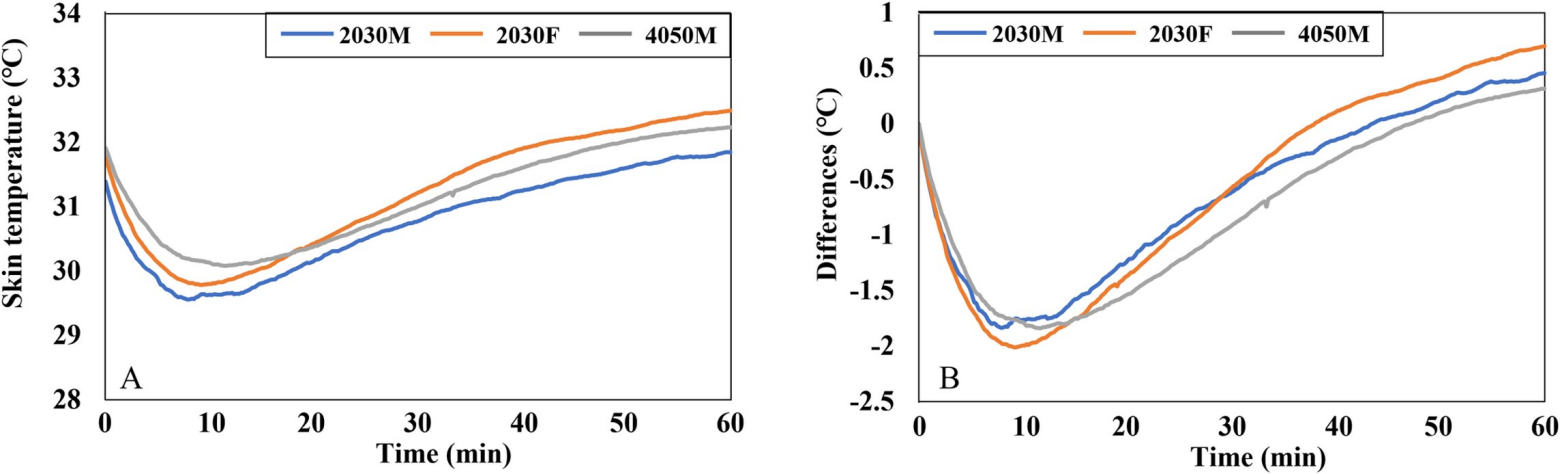
Graphs of MST in the heating conditions. (A) The MST of three groups. (B) The differences in the MST of each group when compared to the MST at the beginning of the experiment (0 min).

In the heating conditions, the MST decreased for the first 10 min despite the heating operation (Fig 11A). This decrease was attributed to the time lag of the system, as it took about 10 min for the engine to unfreeze and the heating system to start functioning effectively, even though the system was turned on at the beginning of the experiments. After this initial period, the MST began to increase when the ambient temperature inside the vehicle rose above 0°C. At the start of the experiment, the MST of the three groups ranged from 31.4°C to 31.9°C. It then dropped by approximately 30°C for 10 min and steadily increased for the remaining time. The MST at the end of the experiment was measured at 31.9°C for 2030M, 32.5°C for 2030F, and 32.2°C for 4050M. Fig 11B describes the change in MST from the beginning of the experiment, showing similar patterns across all three groups. Specifically, the MST of 2030F experienced the greatest decrease during the first 10 min and the greatest increase for the remaining 50 min.

As indicated in Table 5, significant gender differences in MST were observed for the 40–60 min interval. In contrast to the cooling conditions, 2030F exhibited an MST approximately 0.62°C higher than 2030M. On the other hand, similar to the cooling conditions, no significant differences based on age were found during the experiments.

**Table 5:** The results of the t-test for gender and age differences in MST during the heating conditions.

Time (min)	Gender difference (2030M-2030F)		Age difference (2030M-4050M)	
	t-value	p-value	t-value	p-value
0	-1.36	.184	-1.98	.058
5	-0.89	.382	-2.03	.052
10	-0.61	.525	-1.66	.108
15	-0.78	.441	-1.16	.257
20	-0.94	.354	-0.75	.458
25	-1.05	.305	-0.59	.557
30	-1.43	.164	-0.70	.488
35	-1.85	.075	-0.87	.392
40	-2.35	.026**	-1.22	.233
45	-2.28	.030**	-1.38	.179
50	-2.24	.033**	-1.54	.134
55	-2.33	.027**	-1.46	.154
60	-2.46	.020**	-1.47	.152

**p<0.05 (= The differences are statistically significant.)

**Fig 12:**
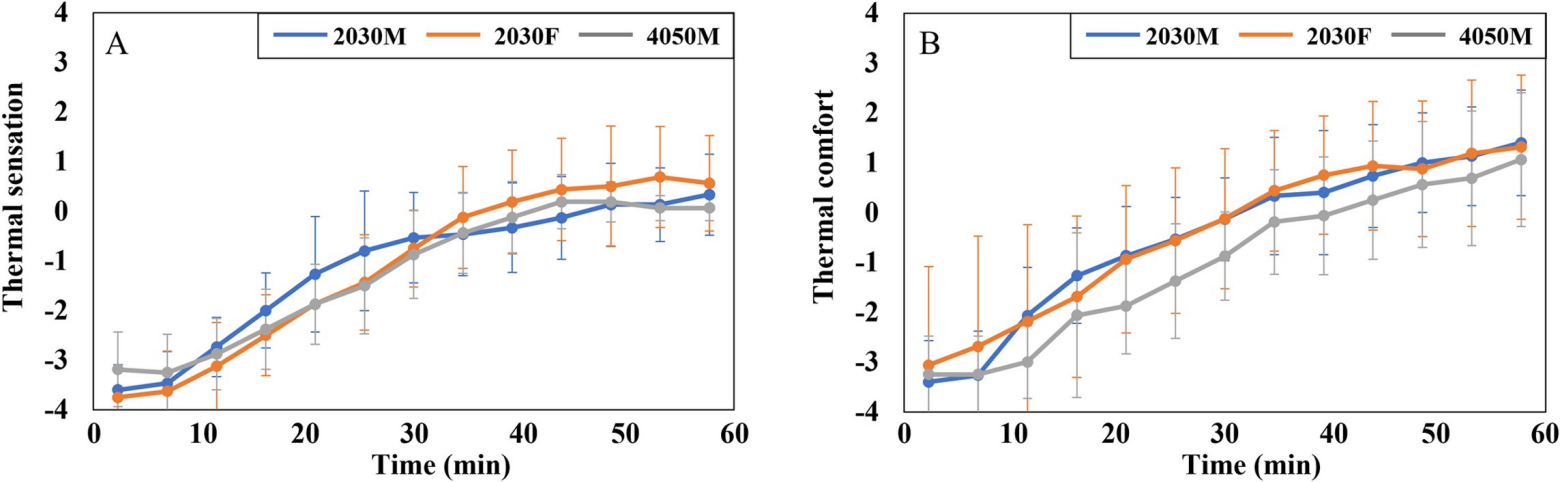
Graphs of psychological responses of three groups in the heating conditions. (A) The mean overall TS. (B) The mean overall TC. The graphs display the mean values and standard deviations for each variable.

The overall TS and TC of the three groups are presented in Fig 12. Initially, the overall TS started at “very cold” (scale -4) and gradually reached a neutral level (scale 0) at approximately 45 min, following a steady increase (Fig 12A). In the beginning, 2030F reported the lowest TS, while 4050M had the highest. However, by the end of the experiments, the TS of these two groups had reversed. The overall TC showed improvement as the TS increased (Fig 12B). All three groups felt uncomfortable in the first 30 min (below scale 0) but started to feel comfortable after that.

In the heating conditions, no statistically significant gender differences were observed in overall TS and TC. However, there were noticeable disparities based on age in overall TC at 10 min and between 20 to 30 min, as indicated in Table 6. During this period, 4050M experienced significantly lower comfort levels compared to 2030M.

**Table 6:** The results of the t-test for age differences in overall TS and TC during the heating conditions.

Time (min)	Overall TS		Overall TC	
	t-value	p-value	t-value	p-value
0	-1.78	.085	-0.52	.606
5	-0.85	.405	-0.06	.956
10	0.60	.556	3.06	.005**
15	1.33	.193	1.62	.115
20	1.70	.099	2.88	.007**
25	1.79	.084	2.32	.027**
30	1.06	.299	2.40	.023**
35	-0.10	.922	1.30	.202
40	-0.71	.481	1.06	.297
45	-1.28	.212	1.21	.237
50	-0.23	.818	1.06	.296
55	0.35	.730	1.04	.306
60	1.23	.235	0.78	.444

**p<0.05 (= The differences are statistically significant.).

#### Local responses

**Fig 13:**
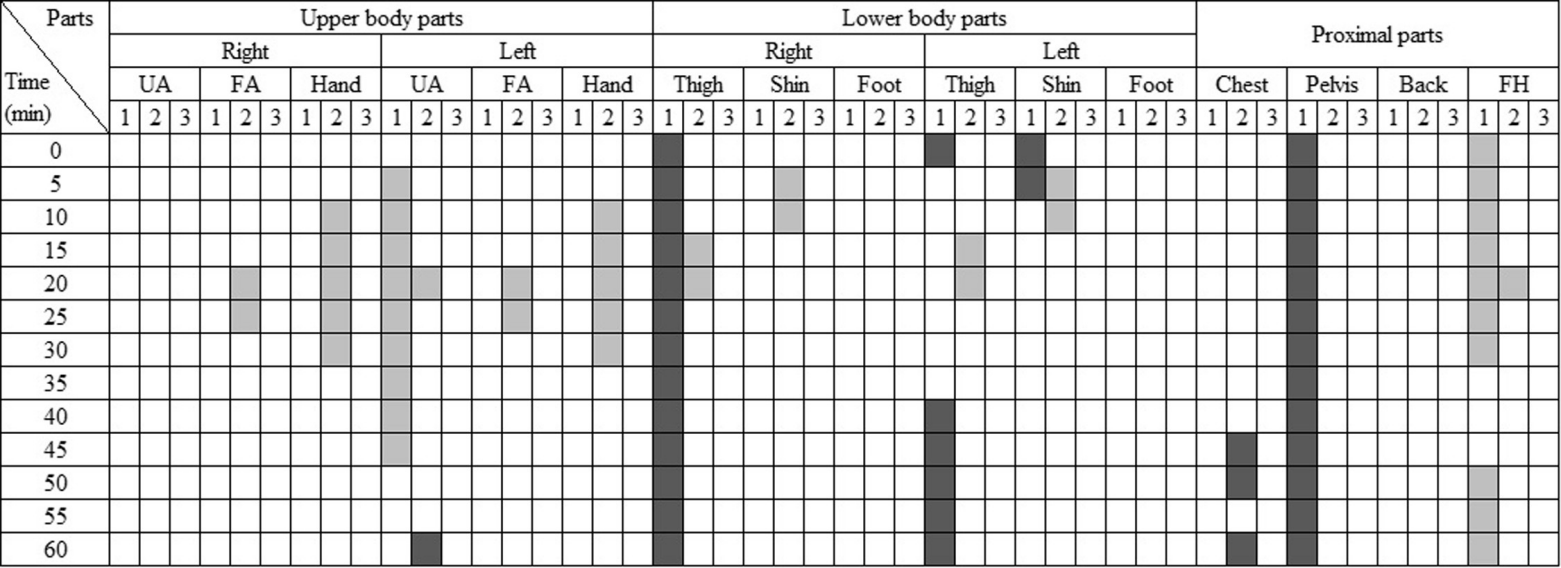
The matrix showing body parts with statistically significant gender differences in the heating conditions. (p < 0.05. UA: upper arm, FA: forearm, FH: forehead. 1: skin temperature, 2: TS, 3:TC. Light color: 2030M had higher skin temperature, TS, TC than 2030F. Dark color: vice versa).

Fig 13 depicts the gender differences in local skin temperature, TS, and TC during the heating conditions. Initially and halfway through the experiments (0–30min), males exhibited higher TS than females in some upper and lower body parts. However, no significant differences were found in skin temperatures and TC, except for the left upper arm and right thigh. In the proximal parts, males had a significantly higher head temperature and lower abdomen temperature than females, but their TS and TC were similar to females.

**Fig 14:**
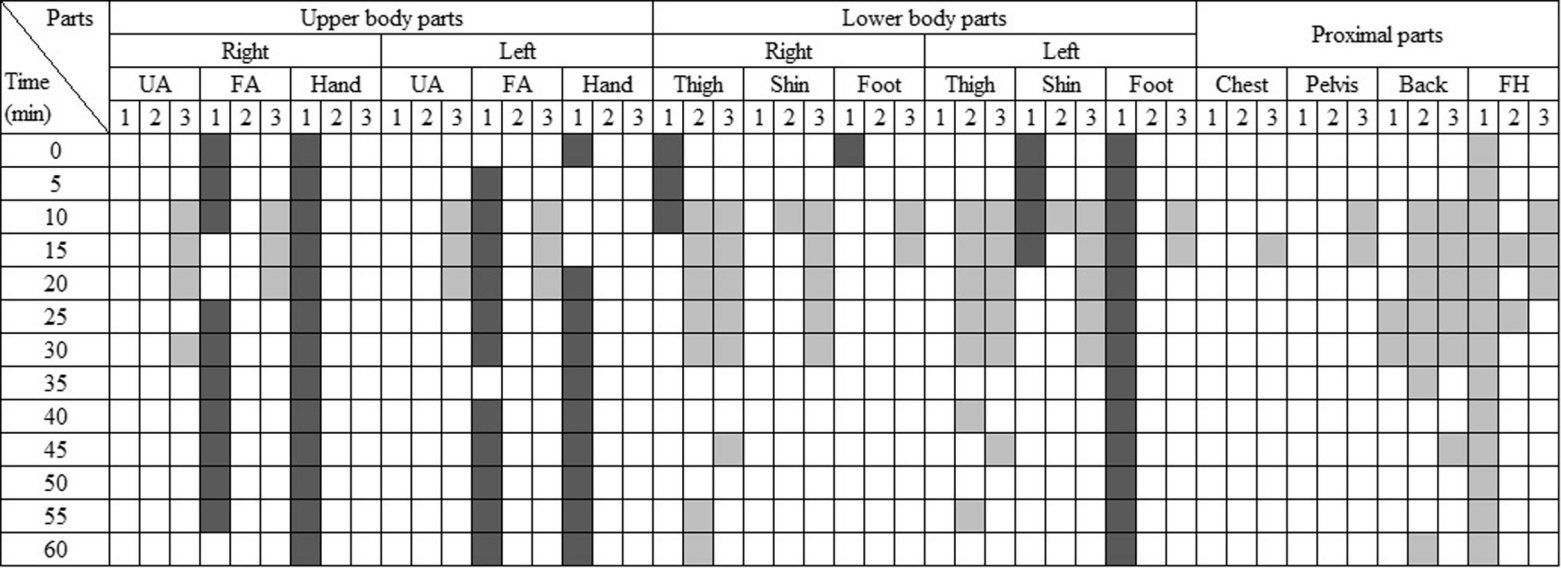
The matrix showing body parts with statistically significant age differences in the heating conditions. (p < 0.05. UA: upper arm, FA: forearm, FH: forehead. 1: skin temperature, 2: TS, 3:TC. Light color: 2030M had higher skin temperature, TS, TC than 4050M. Dark color: vice versa).

Differences in local skin temperatures, TS, and TC between 2030M and 4050M are shown in Fig 14. The skin temperatures of 4050M were higher than those of 2030M at the forearms and hands, but no differences were observed in TS and TC. It indicates that 4050M requires higher temperatures in the forearms and hands to achieve the same level of TC as 2030M in a cold and neutral environment. On the other hand, 2030M exhibited higher TS and TC in the lower body parts and the back compared to 4050M, despite similar skin temperatures. It suggests that 4050M felt cooler and less comfortable even with skin temperatures similar to those of 2030M, indicating a preference for higher temperatures in the lower body parts and the back. At the forehead, the skin temperature of 2030M was higher than 4050M, but TS and TC were similar. It implies that, unlike the lower body parts and back, 4050M can experience similar comfort even with a lower forehead temperature compared to the younger group.

### Gender and age differences in thermal sensation variables

Stepwise multiple regression analysis was conducted to investigate potential differences in variables related to the overall TS based on gender and age. The independent variables included ambient temperature, relative humidity, air speed, MST, proximal temperature, and distal temperature. Previous studies [44–46] found significant differences between proximal and distal temperatures and gender-based disparities in these temperatures. Thus, those two variables were included in this analysis. Proximal temperature represents the average temperature of the chest, abdomen, and back, while distal temperature is calculated by averaging the local skin temperatures on the hands and feet.

Table 7 presents the results of the regression analysis. Under cooling conditions, ambient temperature emerged as the common variable with the greatest impact on overall TS across all three groups. In addition to temperature, relative humidity was found to be significant for 2030M, air speed for 2030F, and humidity and air speed for 4050M. It suggests that overall TS of males is more sensitive to humidity, whereas the TS of females is more influenced by air speed during cooling conditions.

**Table 7:** The significance of variables for overall TS in each condition and group.

Conditions	Group	Variable	β	p-value	Adjusted R^2^
Cooling conditions	2030M	Ta	1.314	0.000	0.968
		RH	0.433	0.001	
	2030F	Ta	0.949	0.000	0.963
		AS	-0.225	0.002	
	4050M	Ta	1.190	0.000	0.997
		RH	0.269	0.000	
		AS	-0.096	0.002	
Heating conditions	2030M	Proximal	0.208	0.000	0.994
		Ta	0.892	0.000	
	2030F	RH	-1.109	0.000	0.992
		AS	-0.259	0.000	
	4050F	RH	-0.825	0.000	0.989
		MST	0.284	0.000	

*Ta = ambient temperature, RH = relative humidity, AS = air speed

During heating conditions, proximal and ambient temperature demonstrated a significant relationship with the overall TS of 2030M. In contrast, humidity and air speed were influential for the overall TS of 2030F and humidity and MST for 4050M.

## Discussion

### Thermal responses in the vehicle

Females and males exhibit various morphological and physiological characteristics, encompassing variances in body shape, physiological regulation, surface-to-volume ratio, muscle mass, and metabolic rate. These gender-related attributes influence the balance of caloric metabolism and give rise to variations in thermal sensation, body temperature distribution, and thermoregulation between females and males [47]. These characteristics were found to contribute to gender disparities in vehicle thermal response in this study. Initially, at the beginning of the cooling condition, both females and males experienced comparable levels of TS (Fig 8A). However, on average, females reported a significantly higher level of overall body comfort compared to males, with a difference of approximately 2 scale units (Fig 8B). It can be attributed to the fact that females have a lower skin temperature, especially in the extremities, as a result of their lower metabolic rate compared to males. This characteristic of females makes them prefer slightly warmer environments [48, 49]. Furthermore, females exhibited the most pronounced change in MST compared to males. Once the air conditioners were activated, the MST of females decreased by approximately 1.5°C (Fig 7B). This finding aligns with the previous study, which confirmed that females experience more rapid changes in MST compared to males, depending on the operative temperature [49].

The HVAC system (air conditioner) was activated immediately after the subjects entered the vehicle when an ambient temperature was approximately 40°C. At that moment (0 min), males experienced significantly higher TS and felt significantly less comfortable than females, despite having similar MST. It indicates a time lag between physiological and psychological reactions in males. Previous research has shown that when individuals cease exercising, the adjustment in temperature perception takes longer to align with changes in the body’s physical indicators, resulting in subjective thermal comfort lagging in regulating the body’s heat balance [50]. However, in contrast to this finding, it was observed that the change in skin temperature appeared later than the thermal sensation in this study. Upon entering the car, individuals immediately felt hot and uncomfortable due to the much hotter environment compared to the pre-conditioned room. However, referring to Fig 7A, it can be observed that the MST of males slightly increased after a few seconds rather than immediately after boarding. This difference in the time lag between our study and the previous one may be attributed to the discrepancy in body movement, as our subjects only walked a short distance when moving from the pre-conditioned room to the vehicle rather than engaging in exercise.

Additionally, males reported significantly lower TC than females at 0 min. According to the previous study, the lower TC experienced by males can be attributed to increased evaporative heat loss. Males had a higher degree of evaporation heat loss compared to females, indicating that they felt hotter and more uncomfortable [48]. However, these disparities disappeared after 5 min, contrasting with previous studies that found continuous significant differences in TC between males and females [32, 51, 52]. The differences in environmental settings and clothing insulation may contribute to the different results between our study and previous studies.

During the cooling conditions, the overall TS of 2030M and 2030F experienced noticeable changes in the first 10 min (Fig 8A). This change was linked to the decrease in MST of more than 1°C (Fig 7A). Interestingly, the decrease in overall TS of 4050M was similar to that of the other two groups (Fig 8A), even though their MST did not drop much (Fig 7A). This relatively small change in MST of 4050M could be attributed to age-related factors. With aging, thermoregulatory function tends to weaken [34]. Therefore, it can be inferred that the set point of skin temperature corresponding to TS may need to be adjusted according to age. This aspect should be further explored, as many physiological thermal comfort models are based on the relationship between skin temperature and thermal comfort.

In both cooling and heating conditions, the ambient temperature reached a neutral level (about 26.5°C) in the middle of the experiments (Fig 5A). When the environment became neutral, the overall TC of all three groups in the cooling conditions stabilized near scale 0 and did not further improve (Fig 8B). In contrast, TC in the heating conditions gradually increased even when the temperature had already stabilized at neutral levels (Fig 12B). The different changing aspects between the two conditions can be attributed to the significant difference in initial comfort. In the cooling condition, comfort at the beginning of the experiment was scale 0 for females and -2 for males, while comfort in the heating condition was the lowest (scale -4) at first for all three groups. Therefore, the lowest comfort in the heating condition at the beginning made subjects feel a neutral environment more comfortable compared to the cooling condition. Then, why subjects felt less comfortable at the beginning of the experiment in the heating condition than in the cooling conditions, even though both conditions are extreme? First, Korea has a humid continental climate with hot summers and dry winters, making people more accustomed to the hot environment than the cold one. Also, the clothing value for heating conditions was 0.85, insufficient to withstand the initial indoor environment of nearly -14°C. Therefore, compared to the cooling condition, the initial comfort level is very low in the heating condition, making the same neutral environment more comfortable.

For some cases, the analysis based on the relationship between local responses and overall responses was done. During cooling conditions, females exhibited lower skin temperatures than males in the upper body, feet, and back, as well as MST (Figs 7A and 9). This observation aligns with findings from previous studies, suggesting that females generally have lower skin temperatures, particularly in the extremities, compared to males [53, 54]. These lower local skin temperatures contributed to the observed lower MST in females. Conversely, under heating conditions, middle-aged males had higher skin temperatures in their forearms and hands in comparison to their younger counterparts (Fig 14). Despite these localized temperature differences, MST remained similar between the two groups (Fig 11). It suggests that the body parts exhibiting temperature differences, such as the forearms and hands, have a relatively limited impact on MST, as confirmed by their lower weighting coefficient in the MST calculation formula. Thus, there were no significant age-related differences in MST despite disparities in local temperatures.

In the case of local comfort, it was found that females reported higher comfort in their lower body (thighs, shins, and feet) compared to males during cooling conditions (Fig 9), but overall comfort was similar between the genders (Fig 8B). Existing studies established that thermal perception varies depending on the body parts [55, 56], with the torso exerting a greater influence on the overall perception of thermal comfort than the limbs or extremities [57]. Therefore, it can be inferred that the elevated comfort levels in the parts other than the torso had no substantial impact on overall comfort for females. In contrast, when younger males had higher comfort in thighs, shins, and back than middle-aged males in heating conditions (Fig 14), they also had higher overall comfort than their counterparts (Fig 12B). In this case, it seems that significant differences in the back substantially affected overall comfort.

Several studies reported the differences in physiological and psychological responses by age. Aging is associated with reduced vasoconstriction intensity and shivering responses in a cold environment. It was shown that the elderly had a lower temperature in their hands, and their ambient temperature regulation was lower than that of young people. Moreover, older people are less sensitive to temperature than younger people, and the rate of perception of changes in the hypothalamus temperature is slow [34, 35, 58, 59]. However, those findings were not confirmed in this study because the target groups for age differences were 2030M and 4050M, not the elderly as in the other studies, and the age gap was not that large.

The results of regression analysis considering various variables and overall TS indicate that ambient temperature commonly influenced the overall TS of all three groups in the cooling condition. This result is consistent with previous studies, which have found that air temperature is the most influential variable among environmental factors [60–63]. Additionally, females demonstrated a heightened sensitivity to air speed, emphasizing the importance of regulating air speed for female passengers in both cooling and heating conditions.

### Potential applications and limitations

This study aimed to investigate the overall and local physiological and psychological responses of different passengers, with a specific focus on examining the influence of gender and age on thermal responses in an actual vehicle. In contrast to previous studies, this study contributes significantly to the field by providing comprehensive insights into the thermal environment and passengers’ responses within a vehicle context. Considering the development of various types of vehicles and the increasing amount of time people spend in them, the creation of a comfortable environment for passengers has become crucial. Moreover, the control of the indoor environment of the vehicle is transitioning from conventional control to individual customized control, allowing for the personalized adjustment of the HVAC system to meet the specific needs of each passenger. By identifying the specific thermal comfort needs related to age and gender and incorporating these differences into the design of the vehicle’s HVAC control, a more comfortable environment can be created. Understanding individuals’ reactions and characteristics serves as a fundamental and essential starting point in achieving this goal.

However, there are several limitations that should be acknowledged. Firstly, the experimental conditions employed in this study were quite extreme. While these conditions are commonly used for testing the HVAC system’s performance in vehicles globally, they may not accurately reflect the typical driving environment experienced by most individuals. Future studies should consider conducting experiments under milder conditions to bridge this gap. Secondly, the exclusion of females in their forties and fifties, based on their physiological characteristics, is another limitation. Additionally, this study focused on a single passenger seated in the driver’s seat, excluding scenarios involving passengers in other seating positions or multiple occupants. These limitations highlight the need for further research to supplement these aspects and obtain more in-depth results and insights into passengers’ physiological and psychological responses within the vehicle.

## Conclusion

In this study, the subjects were exposed to cooling and heating conditions under a controlled vehicle environment to examine the gender and age differences in MST, TS, and TC. The major findings of this study are as follows:

- During the cooling conditions, the MST of 2030F was consistently lower than that of 2030M. Despite the significant differences in MST, gender differences in overall TS and TC were found only in the very transient environment at the beginning (0–5 min). Afterward, both groups had similar TS and TC for the rest of the time.
- Generally, 2030M and 4050M had similar physiological and psychological responses during the experiments. The overall TS of 4050M showed a similar decrease to 2030M and 2030F, even though their MST did not drop much, unlike young groups.
- In the heating conditions, significant gender differences in MST were found after the environment became stable (40–60 min). 2030F had significantly higher MST than 2030M during that period, opposite to the cooling conditions. However, their overall TS and TC were similar without significant differences.
- In the case of male groups, there were significant age differences only in overall TC in the middle of the experiments (10, 20–30 min). During that time, the comfort of 4050M was significantly lower than that of 2030M.

The findings of this study underscore the significance of considering gender and age as critical determinants in understanding the varying physiological responses and thermal comfort experienced by passengers in a vehicle environment. Furthermore, the investigation reveals that variations in local responses do not necessarily translate into corresponding disparities in the overall thermal response. These results hold potential implications for enhancing the comprehension of thermal comfort among vehicle occupants. They also could serve as fundamental data for subsequent research endeavors aimed at devising thermal prediction models and system control logic, ultimately striving to provide a more comfortable and personalized environment for passengers.

## Abbreviations

2030M: Males in their twenties and thirties
2030F: Females in their twenties and thirties
4050M: Males in their forties and fifties
MST: Mean skin temperature
TS: Thermal sensation
TC: Thermal comfort
